# High-LET radiation induces large amounts of rapidly-repaired sublethal damage

**DOI:** 10.1038/s41598-023-38295-3

**Published:** 2023-07-11

**Authors:** Francisco D. C. Guerra Liberal, Shannon J. Thompson, Kevin M. Prise, Stephen J. McMahon

**Affiliations:** Patrick G Johnston Centre for Cancer Research, Queen’s University Belfast, 97 Lisburn Road, Belfast, BT9 7AE UK

**Keywords:** Computational biology and bioinformatics, Radiotherapy, Computer modelling, Biological physics

## Abstract

There is agreement that high-LET radiation has a high Relative Biological Effectiveness (RBE) when delivered as a single treatment, but how it interacts with radiations of different qualities, such as X-rays, is less clear. We sought to clarify these effects by quantifying and modelling responses to X-ray and alpha particle combinations. Cells were exposed to X-rays, alpha particles, or combinations, with different doses and temporal separations. DNA damage was assessed by 53BP1 immunofluorescence, and radiosensitivity assessed using the clonogenic assay. Mechanistic models were then applied to understand trends in repair and survival. 53BP1 foci yields were significantly reduced in alpha particle exposures compared to X-rays, but these foci were slow to repair. Although alpha particles alone showed no inter-track interactions, substantial interactions were seen between X-rays and alpha particles. Mechanistic modelling suggested that sublethal damage (SLD) repair was independent of radiation quality, but that alpha particles generated substantially more sublethal damage than a similar dose of X-rays, $RB{E}_{SLD}>2.8$. This high RBE may lead to unexpected synergies for combinations of different radiation qualities which must be taken into account in treatment design, and the rapid repair of this damage may impact on mechanistic modelling of radiation responses to high LETs.

## Introduction

The differing Relative Biological Effectiveness (RBE) of high Linear Energy Transfer (LET) radiations is well-established for numerous biological endpoints. As heavy charged particles (such as protons and carbon ions) slow, they deposit their energy more densely, leading to greater damage for a given dose compared to X-rays, including elevated rates of mutations, chromosome aberrations, and cell killing^1–3^. This elevated RBE is a key advantage of ion-based therapies, as tailoring elevated RBE to tumour regions may improve clinical outcomes^4,5^.

Typically, increasing LETs are associated with a steepening of the dose response, interpreted as an increase in the α term in the Linear-Quadratic (LQ) survival model. Elevated α values represent increasing single-hit lethality^6^, so the increase in RBE with LET is interpreted as an increase in lethality per incident track. This has been linked to increased damage complexity and high levels of damage around individual tracks^7,8^. This complexity is also associated with the observation of slower repair kinetics of DNA damage as LET increases^9–11^.

However, the exact mechanisms underpinning these effects remain unclear. Damage complexity is a broadly defined term in radiotherapy, typically used to refer to lesions which consist of multiple individual damages. Double Strand Breaks (DSBs) are one of the most important such damages, representing a combination of two nearby strand breaks on opposite strands. More complex lesions can also be formed, some of which are so-called ‘complex DSBs’ associated with additional base and strand damages, as well as non-DSB clusters of damages^12^. These latter can, in some cases, be converted into further DSBs through the failure of repair processes^13,14^.

A range of models have been developed to relate LET and RBE applying varied assumptions about whether increasing LET leads to a greater yield of DSBs, a greater complexity of DSBs, or both, but to date it has not been possible to clearly distinguish the relative contributions of yield, complexity, and other effects^15,16^. The higher dose per incident particle further obscures these effects, as clustering of damage around a small number of distinct tracks effectively increases α and decreases β even if particle-induced damages are not more lethal individually.

This is significant because in addition to ‘lethal’ damage which is alone sufficient to kill cells, radiation also induces so-called ‘sub-lethal’ damage (SLD). SLD refers to types of damage which can individually be repaired by cells, but which may interact with other sub-lethal damage to form lethal damage—such as two DSBs being incorrectly joined together to form chromosome aberrations. This underlies the quadratic component of LQ radiation response curves, underpinning the effects of fractionated radiotherapy^6^.

One method to distinguish between these mechanisms is through mixed-field exposures, combining radiations with significantly differing LETs. Such studies are relatively uncommon compared to single-field studies, due to their additional experimental complexity and the limited clinical applicability as most patient treatments are delivered with a single modality. However, recent interest in combining different radiation qualities (including X-rays and alpha particle emitting radionuclides^17,18^, X-ray and protons^19,20^, and multi-ion treatments^21^) have provided an impetus to explore this in greater detail.

Published data on this topic has reported mixed results. A combination of alpha particles and X-rays have been widely investigated, but various studies suggest no interaction^22–24^ or significant interactions^25–28^, together with differing effects on the complexity of repair^29,30^ when these radiations are combined. It is thus challenging to mechanistically interpret this data either in terms of the overall yield of SLD or its repair lifetime, factors which are important in any attempt to understand mixed-field treatments.

This also contributes to uncertainties in the modelling of radiation-induced damage and repair more generally. Many models make assumptions about how repair and survival is affected by damage ‘complexity’, often expressed in terms of the clustering of multiple DSB and non-DSB damages in close proximity, to explain the LET dependence of radiation response^16,31–33^. However, there is disagreement about the role of complexity on both repair and survival, introducing considerable uncertainty into model comparisons. A better understanding of SLD repair at different LETs would also help to mitigate this issue.

In this work, we tested combinations of alpha particle and X-ray exposures delivered with varying doses and temporal separations, to map out the characteristics of SLD induced by both radiations. Based on this, we implemented a simplified mechanistic model to explore how these results could be interpreted in terms of the repair of individual lesions.

## Methods

### Cell culture

PC-3 prostate bone metastasis and U2OS osteosarcoma cells were obtained from the American Type Culture Collection (ATCC, Manassas, Virginia, USA). PC-3 cells were propagated in RPMI-1640 medium (11875093, Gibco), U2OS cells were propagated in high glucose DMEM (10313021, Gibco), both supplemented with 10% fetal bovine serum (102701106, Gibco) and 1% penicillin–streptomycin (10378016, Gibco). All cultures were incubated at humidified 37 °C in 5% CO_2_.

### Irradiation sources

X-ray irradiations were performed using an X-RAD 225 radiation source (Precision X-ray Inc. USA) at 225 kV, 13.3 mA, at a dose rate of 0.59 Gy/min. For alpha particle irradiation, Mylar dishes with a thickness of 0.9 μm were placed 2.9 mm from a 50 × 50-mm planar ^241^Am alpha source, with a dose rate of 1.57 Gy/min. Incident average energy at the cells was 2.88 $\pm$ 1.04 MeV with LET 129.3 $\pm$ 15.2 keV/μm, as previously described^34^.

### Clonogenic assay

For single fractions of X-rays, cells were seeded into 6-well plates with an optimal cell density to ensure the formation of colonies. On the following day samples were then exposed to single fractions of X-rays ranging from 0 to 8 Gy. After irradiation cells were incubated for 8 days. And colonies were stained, using a solution of 0.5% crystal violet (Sigma-Aldrich, UK) in 90% methanol.

Alternatively, for single fractions of alpha particles from 0 to 2 Gy, or mixed field sequential treatment with equal doses of X-rays and alpha particles, with 1 h between fractions, 1 × 10^5^ cells were seeded onto sterile Mylar dishes and incubated to adhere overnight.

Mixed fields irradiations were carried out with both X-ray followed by alpha particle exposures and vice-versa. Delivered doses were 0.25 Gy + 0.25 Gy, 0.5 Gy + 0.5 Gy, 0.75 Gy + 0.75 Gy, 1 Gy + 1 Gy and 2 + 2 Gy. Immediately after irradiation, cells were reseeded in triplicate into 6-well plates at an optimal cell density to ensure the formation of colonies. Cells were fixed 8 days post-irradiation, using a solution of 0.5% crystal violet (Sigma-Aldrich, UK) in 90% methanol. Colonies were manually counted with a 50-cell exclusion criterion was used. Clonogenic Survival Fractions (SF) were calculated as the ratio of the number of colonies in the irradiated flask to the number of seeded cells, corrected for the platting efficiency of control cells. Mean and standard deviations were calculated from three independent experiments. Dose response-curve data was fitted to the linear quadratic equation

1 $$\begin{array}{*{20}c} {S = e^{{ - \alpha D - \beta D^{2} }} } \\ \end{array}$$

To predict survival for a purely additive interaction between X-rays and alpha particles, the following modification to the linear quadratic equation was used

2 $$\begin{array}{*{20}c} {S = e^{{ - \alpha_{A} D_{A} - \alpha_{x} D_{x} - \left( {D_{A} \sqrt {\beta_{A} } + D_{x} \sqrt {\beta_{x} } } \right)^{2} }} } \\ \end{array}$$

where the subscripts $A$ and $x$ represent alpha particles and X-rays, respectively^35,36^.

### Sublethal damage repair

To quantify the impact of time between radiation fractions on response, 1 × 10^5^ cells seeded onto sterile Mylar dishes were exposed to two fractions of either X-rays, alpha particles, or mixed field combinations as described above. The dose for each radiation quality was chosen to give approximately 10% survival. This was 3 Gy for X-rays and 0.75 Gy for alpha particles. After exposure to the first fraction cells were returned to an incubator until the second fraction. Time intervals between fractions varied from 15 min to 6 h. Immediately after exposure to the final fraction, cells were reseeded in triplicate into 6-well plates at an optimal cell density to ensure the formation of colonies.

The resulting survival was then quantified as described above. The Lea-Catcheside dose protraction factor for 2 fractions was then used to characterise this data. Survival was predicted as

3 $$\begin{array}{*{20}c} {S = e^{{ - \alpha D - \beta GD^{2} }} } \\ \end{array}$$

where the dose protraction factor, G, is

4 $$\begin{array}{*{20}c} {G = \frac{1}{\lambda t}\left[ {1 - \frac{1}{\lambda t}\left( {1 - e^{ - \lambda t} } \right) + \frac{{{\text{e}}^{ - \lambda T} }}{2\lambda t}\left( {1 - e^{ - \lambda t} } \right)^{2} } \right]} \\ \end{array}$$

with $\lambda$ the cell’s repair constant, $t$ the fraction delivery time, and $T$ the inter-fraction interval^37,38^.

To estimate $RB{E}_{SLD}$, we adapted the approach underlying the LQ and Lea-Catcheside models, where the quadratic (β) term in the LQ is related to the interaction between the yields of sublethal damage for each exposure. This implies that the magnitude of recovery from acute exposures to those with long time separations is proportional to the yield of SLD.

In this case, we consider an example of cells exposed to two fractions, one fraction of X-rays followed by one fraction of alpha particles, with a sufficiently long interval between the two fractions that repair has completed. The resulting predicted survival is

5 $$\begin{array}{*{20}c} {S_{\infty } = e^{{ - \alpha_{x} D_{x} - \beta_{x} D_{x}^{2} }} e^{{ - \alpha_{A} D_{A} - \beta_{A} D_{A}^{2} }} } \\ \end{array}$$

which is the standard LQ-predicted survival for cells irradiated at a sufficiently long interval that there is no interaction between damage from the two fractions. For acute exposures, this then becomes

6 $$\begin{array}{*{20}c} {S_{0} = e^{{ - \alpha_{x} D_{x} - \beta_{x} D_{x}^{2} }} e^{{ - \alpha_{A} D_{A} - \beta_{A} D_{A}^{2} }} e^{{ - \gamma SLD_{x} SLD_{A} }} } \\ \end{array}$$

where the first part of the expression is as in Eq. (5), and the final term represents an interaction between SLD yields for the X-rays and alpha particles. In particular, $SL{D}_{x}$ and $SL{D}_{A}$ represent the yields of sublethal damage for X-rays and alpha particles, and $\gamma$ is a fitting constant. This follows the underlying model as applied in the derivation of the Lea-Catcheside model, but incorporates that X-rays and alpha particles may induce different yields of sublethal damage.

The specific contribution of the sublethal damage interaction can be separated out by taking the log of survival for acute and well-separated fractions, and taking their difference to obtain:

7 $$\begin{array}{*{20}c} {\ln \left( {S_{\infty } } \right) - \ln \left( {S_{0} } \right) = \gamma SLD_{x} SLD_{A} } \\ \end{array}$$

This gives a measure of the relative recovery for sequential exposures to X-rays and alpha particles.

To determine $RBE_{SLD}$, we can perform the same analysis for two fractions of X-rays, with the assumption that $SLD_{x}$ is a constant. The difference in acute and protracted survivals for two X-ray exposures is then $\gamma SLD_{x} SLD_{x}$. If we assume that $\gamma$ is a cell-line specific constant, we can take the ratio of these terms for the X-ray + alpha exposures and X-ray + X-ray exposures to calculate the ratio $\frac{{SLD_{A} }}{{SLD_{x} }}$—that is, the relative yield of sublethal damage induced by our fractions of alpha particles and X-rays. The RBE is then given by this ratio of sublethal damage divided by the ratio of doses delivered, that is:

8 $$\begin{array}{*{20}c} {RBE_{SLD} = \frac{{SLD_{A} }}{{SLD_{x} }}\frac{{D_{x} }}{{D_{A} }}} \\ \end{array}$$

For the purpose of this work, all alpha particle RBEs are quoted with reference to the 225 kVp irradiation source.

### Immunofluorescence

Cells were irradiated with 2 Gy of X-rays or alpha particles. Following irradiation, cells were fixed in 50:50 methanol-acetone solution and permeabilized (0.5% of Triton X-100 in PBS) at predetermined time points before being blocked in blocking buffer (5% FBS, and 0.1% Triton X-100 in PBS) and stained with 53BP1 primary antibody (1:5000) (NB100-304, Novus Biologicals, USA) for 1 h before being washed three times and stained with Alexa Flour 568 goat anti-rabbit IgG secondary antibody (1:2000) (A21429, Life Technologies, USA) in the dark for 1 h. Following staining, the cells were washed three times and mounted onto microscope slides using the Prolong Gold antifade reagent with DAPI (P36930, Invitrogen, USA). Foci were manually counted from the whole nucleus of 50 randomly selected cells on each sample with a Zeiss Axiovert 200 microscope (Carl Zeiss), using a × 63 objective. Mean counts and standard deviations were calculated from three independent experiments. All presented data is corrected for number of foci present in unirradiated cells. For repair kinetics analysis, foci data were then fit with an exponential decay in GraphPad Prism 7, $N=\left({N}_{0}-P\right){e}^{-kt}+P$, where ${N}_{0}$ represents the initial number of 53BP1 foci, $P$ represents the residual damage, and $k$ is the rate of DSB repair.

### Simple DNA damage model

TOPAS-nBio 3.6.1^39^ was used to simulate the irradiation of a simplified nucleus to estimate DSB yield. The model makes use of a small 2.5 µm radius nucleus which is uniformly sensitive to radiation. This has been shown elsewhere in the literature to reproduce trends in DNA damage yields without requiring a detailed DNA geometry^40,41^, in contrast to other models which made use of a larger nucleus with other geometric assumptions^16,42^.

The particle spectrum of the 225 kVp source at a depth of 2 mm in water was simulated, and used to irradiate the model nucleus to a dose of 1 Gy. The position and energy deposit of each interaction was recorded. Energy deposition events were randomly converted to single-strand breaks (SSBs) using a probability proportional to the energy deposited per event. The average energy deposit per SSB was calculated by dividing the total energy deposited in the nucleus by the number of strand breaks, assuming 1 Gy induces 1000 SSBs^43^. SSBs were randomly assigned to a strand, with two strand breaks on opposite strands within 3.2 nm clustered into DSBs. This was averaged across 10,000 SSB sampling repeats to find the mean DSB yield. Using an average energy per SSB of 0.41 keV produced a DSB yield in line with 35 DSBs/Gy associated with low LET exposures^44^.

1 Gy irradiations using proton and helium ions with energies ranging from 0.3 to 67 MeV/u were then simulated using these parameters. For each condition 10–12 independent runs with different random seeds were simulated, and the mean and standard deviation of DSB yields per Gy were calculated. $RB{E}_{DSB}$ for our source was then estimated from the corresponding LET.

### Mechanistic survival modelling

The interaction between DSB complexity, DNA repair, and survival remains an outstanding question in modelling radiation responses, particularly with varying LET. Here, using the Medras model, we tested the assumption that there is no LET-dependence on the complexity of damage repair as it relates to sublethal damage repair^44–46^. Medras is a mechanistic model of DNA damage response, which simulates radiation responses beginning from a distribution of DNA DSBs within the nucleus. It assumes that initial misrepair events relevant to sublethal damage repair are independent of LET and any associated break complexity, which we sought to test against these data. For the purposes of this analysis, both cell lines were modelled as spherical human cells, with a radius of 4.32 μm, as used in a previously published RBE analysis^46^.

For X-ray exposures, DSBs positions were randomly sampled from a uniform distribution throughout the nucleus, with a yield of 35 DSBs/Gy. For alpha particle exposures, DSBs are deposited around tracks, with a break probability density function based on the radial energy distribution around the track, which was calculated using Geant4-DNA^47,48^. For this work, we have incorporated an LET-dependent yield of DSB per unit dose, as used in many other radiobiological models. To achieve this, in these models the reference X-ray DSB yield was scaled based on the $RB{E}_{DSB}$ predicted from the simple nucleus model described above, the results of which are shown below in Fig. 4. For both X-ray and alpha particle exposures, breaks were randomly assigned as either ‘simple’ or ‘complex’ based on a purely probabilistic function, with a complex break rate of 0.43, based on previous fits to X-ray data^45^.

DSB repair was then stochastically calculated as a function of time. Each DSB comprised a pair of two free ‘ends’. Each DSB end can repair with any other free DSB end, with a repair rate per unit time given by $\zeta \propto {e}^{-\frac{{r}^{2}}{2{\sigma }^{2}}}$. $r$ is the spatial separation between the two break ends and $\sigma$ is a model fitting constant describing the dependence of repair on end separation. This process is iterated until all break ends had been repaired. Breaks are considered correctly repaired if their two ends joined to their matching partners, and misrepaired if they joined ends from different breaks. This approach has been previously shown to accurately reproduce misrepair at a range of doses and conditions^44^.

500 independent damage distributions were generated for each single-field exposure, and 1000 for each mixed-field exposure. For single-fraction exposures, all breaks were generated at a single time. For fractionated exposures, damage distributions were generated for each fraction, and then repair was simulated with the second exposure occurring at varying intervals after the first. This reduced the statistical variability in mixed field exposures due to potentially greatly varying contributions of alpha and X-ray doses to the total DSB yield, without affecting the expected behaviour. Repair was simulated 3 times for each damage distribution. For each condition, the misrepair rate was calculated as the average across all simulations.

In full cell survival calculations, Medras predicts the probability of survival as a combination of both the yield of lethal aberrations and mutations (driven by misrepair events) as well as the contribution of active pathways which prevent proliferation, such as apoptosis and senescence. As this work represents only a limited sampling of responses for this cell line, a simplified survival model was used, given by:

9 $$\begin{array}{*{20}c} {S\left( t \right) = S_{\infty } e^{{ - k\left( {M\left( t \right) - M_{\infty } } \right)}} } \\ \end{array}$$

Here, $S(t)$ is the fraction of cells surviving at time $t$, and ${S}_{\infty }$ is the fraction of cells surviving when the two fractions are delivered sufficiently far apart that there is no interaction between them. $M(t)$ and ${M}_{\infty }$ are similarly the rates of misrepair at time intervals of $t$ and long separations respectively, and $k$ is the probability that a misrepair event leads to cell death (e.g. through the formation of a lethal chromosome aberration, as used in Medras^45^).

By taking the difference between the misrepair rates at long separations (representing individual well-separated fractions), and more closely occurring fractions, the difference in the expected number of lethal aberrations can be calculated, and thus the added probability of cell killing due to such an event (assuming such events are Poisson distributed). ${S}_{\infty }$ and $k$ were fit for each exposure to determine the relationship between misrepair and lethality. This was achieved using Scipy’s optimize_curve_fit routine^49^.

### Foci kinetics modelling

The impact of DSB clustering on foci kinetics was also modelled. It was assumed that radiation induced damage was distributed in a series of clusters. Each cluster contains a number of distinct individual DSBs, but is assumed to be detected as a single foci. DSBs are then assumed to be individually repaired with simple exponential kinetics, but the cluster is only cleared once all its constituent DSBs have been repaired.

This was simulated by distributing (on average) $N$ initial damage clusters throughout the nucleus, each containing $F$ DSBs. For X-rays, damage was assumed to be sparsely distributed, so $F=1$, and thus $N$ is equal to the total number of DSBs. For alpha particles, it is assumed that the total number of DSBs is given by the $RB{E}_{DSB}$ relationship predicted from the Monte Carlo modelling (see above and Fig. 4, below), but that each cluster may contain more than 1 DSB. The number of breaks per cluster were randomly sampled with a Poisson distribution, taking into account the total number of DSBs and the path length of the alpha particle through the nucleus for each cluster. As in the misrepair model, breaks were randomly designated as simple or complex, with repair coefficients taken from published Medras values^45^.

The average number of visible DSB clusters was then calculated as a function of time based on the probability of all of the individual foci within a cluster being repaired. These values were then fit simultaneously to the measured foci yields of X-rays and alpha particles by varying the initial yield of observed foci in the X-ray irradiation, the fraction of persistent X-ray foci at 24 h, and the number of clusters in the alpha particle exposure. Fitting was performed using Scipy’s optimize_curve_fit routine, to determine if it could accurately reproduce the observed foci kinetics.

## Results

### DSB repair kinetics

**Figure 1:**
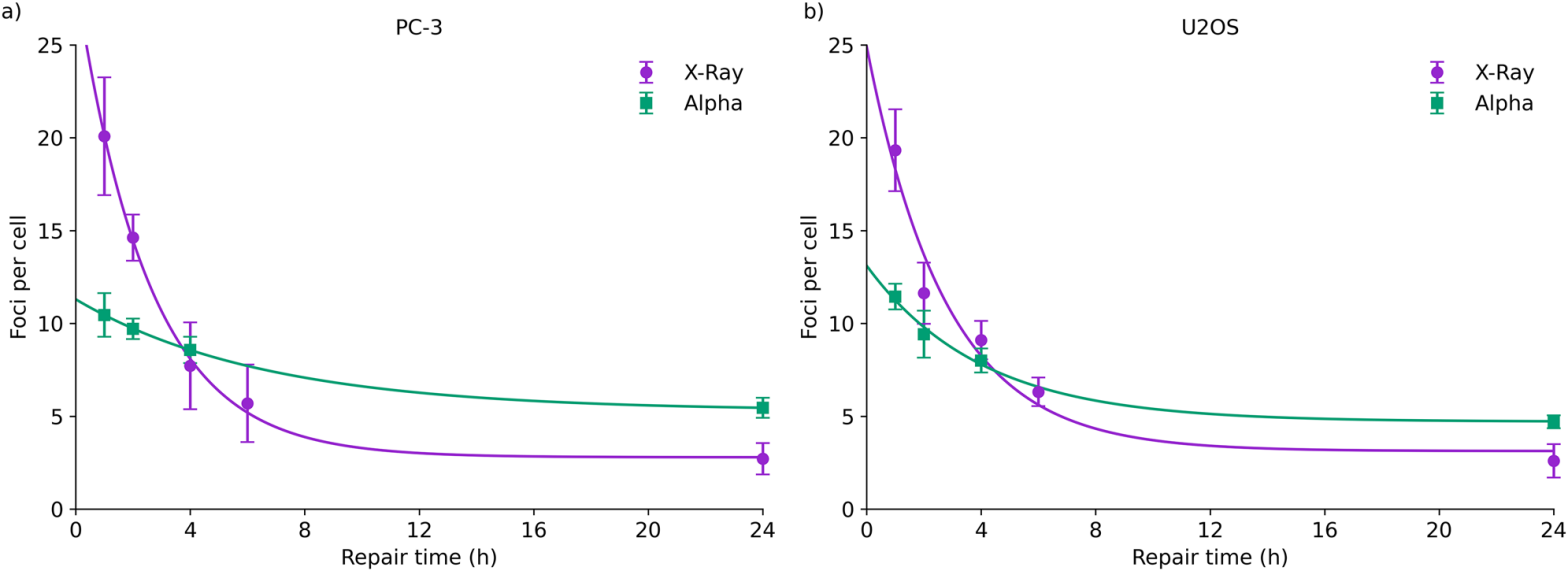
DNA damage assessed using 53BP1 immunofluorescence for PC-3 (left) and U2OS (right) cells, following 2 Gy irradiation with X-rays or alpha particles. Both cell lines show similar trends, with a larger number of rapidly repaired X-ray induced foci, compared to fewer alpha-induced foci which are repaired much more slowly. Points are mean and standard deviation of measured data (N = 3); lines are exponential fits.

Figure 1 shows the kinetics of DSB foci (53BP1) repair as shown by immunofluorescence for PC-3 and U2OS cells, exposed to 2 Gy of X-rays or alpha particles. X-ray exposures present characteristic DSB repair curves, with exponential repair kinetics and a degree of persistent damage at later timepoints. Similar levels of foci are observed in both cell lines, despite their different tissues of origin, morphology, and ploidy.

In contrast, alpha particle irradiations show fewer initial foci, with an approximately 40% reduction in foci at 1 h compared to X-rays. The clearance of these foci is much slower, however, with more foci persisting in alpha-irradiated cells than X-ray irradiated cells at times later than 6 h, and significantly more persistent foci at the 24 h time point (87% of X-ray induced foci at 1 h are cleared by 24 h in both cell lines, compared to only 58% and 47% of alpha-induced foci in U2OS and PC-3 respectively, p < 0.02 by t-test between 24 h foci in X-ray and alpha-exposed cells for each cell line).

This appears to suggest that alpha particles induce a smaller number of more complex DSBs, which take longer to repair. However due to the limited imaging resolution of immunofluorescence assays, it is not possible to determine directly from this data how many DSBs are present in any given immunofluorescent focus.

### Mixed-field clonogenics

**Figure 2:**
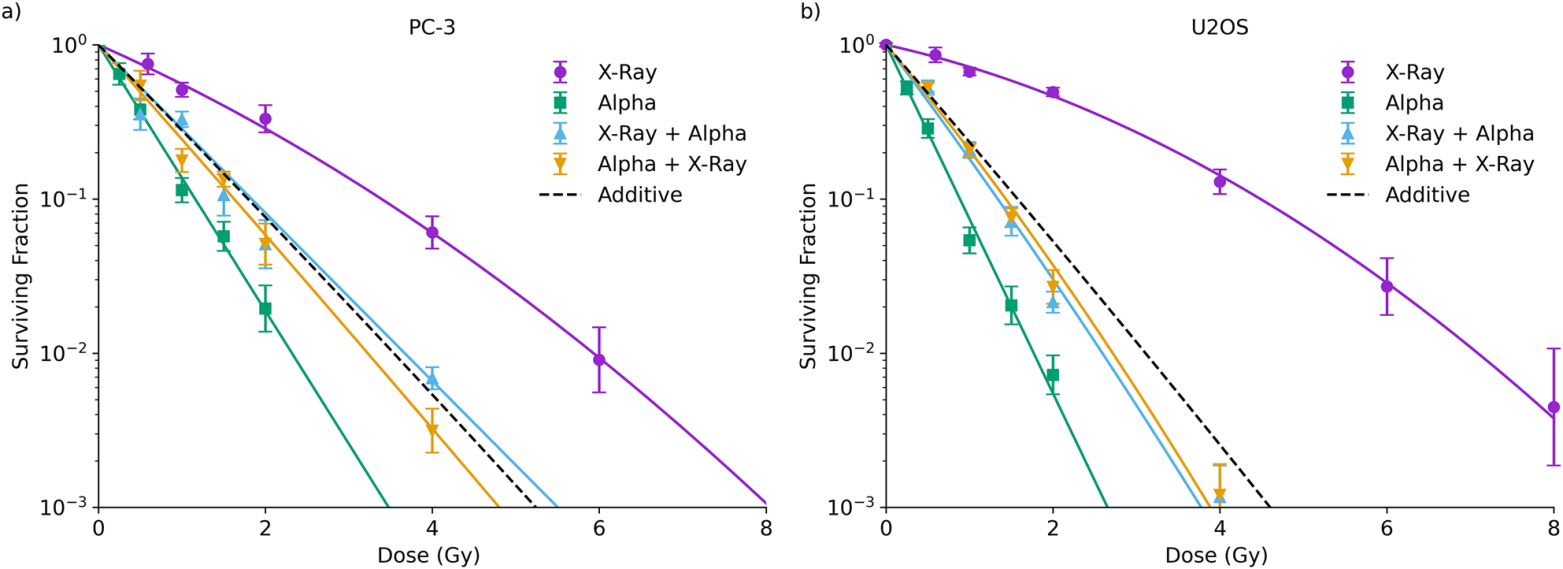
Clonogenic survival following single- and mixed-field irradiation in PC-3 (left) and U2OS (right) cells. Mixed-field irradiations were delivered as equal doses at an interval of 1 h. Single-field survival curves appear as expected, with a linear-quadratic dose response to X-rays, and a more sensitive linear dose response to alpha particles. In mixed-field irradiations, no significant difference was seen based on the ordering of the radiation exposures (p > 0.12), and the observed responses are in good agreement with predictions of a purely additive model. Points are mean and standard deviation of measured data (N = 3); solid lines are linear-quadratic fits; dashed line is an additive model as described in the “Methods”.

Figure 2 shows the clonogenic survival of both cell lines as a function of dose following exposure to either single- or mixed-field irradiations. Single-field dose response curves show typical linear quadratic responses for X-ray irradiation, and a steeper, linear, response to alpha particle irradiations. PC-3 cells show slightly greater sensitivity to X-rays and lower sensitivity to alpha particles compared to U2OS giving a lower alpha particle RBE ($RB{E}_{{D}_{10}}=3.0\pm 0.3$ and $4.9\pm 0.7$ for PC-3 and U2OS respectively).

In both cell lines, combinations of equal doses of X-rays and alpha particles separated by 1 h gave equal effects regardless of which radiation type was delivered first (p > 0.12 by extra-sum-of-squares F test). In both cases, these were in good agreement with a simple additive model, assuming no interaction between fractions (dashed line). This suggests that, contrary to what might be expected from the foci data presented in Fig. 1, there is a large amount of persistent sub-lethal damage at this timepoint which would lead to greater synergy between exposures.

### Sublethal damage repair

**Figure 3:**
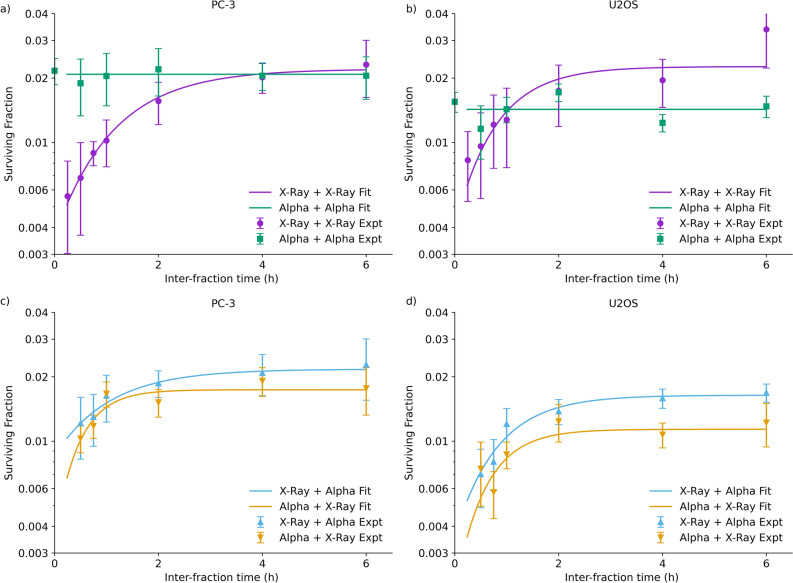
Sublethal damage repair curves for PC-3 (left) and U2OS (right) cells following exposure to two identical fractions separated by 15 min to 6 h (2 × 3 Gy X-rays or 2 × 0.75 Gy alpha particles, top) or mixed exposures (bottom, 3 Gy X-rays and 0.75 Gy alpha particles). X-ray exposed cells show clear recovery, while alpha-exposed cells show negligible effects. In contrast, repair is seen in mixed exposures of X-rays and alpha particles, regardless of the initial radiation, suggesting sublethal damage recovery occurs even following alpha exposures. Points are mean and standard deviation of measured survival (N = 3); solid lines are fits as described in the “Methods”.

To investigate this further, the dependence of cell survival on the interval between fractions of different radiations was investigated, shown in Fig. 3. As expected, clear SLD repair is seen in the X-ray irradiated cells, with survival increasing by a factor of 4–5 when 6 Gy is delivered in two fractions at a 6 h interval, compared to a single dose. By contrast, for alpha particle exposures, no sublethal repair is seen when 1.5 Gy is delivered in two fractions, with a slope which not significantly different from zero in either cell line (p > 0.9, F-test comparing linear regression slope of survival in each cell line to zero).

This appears to agree with Fig. 1, showing greater SLD repair for X-rays than alpha particles. However, as alpha particles deliver their dose through a relatively small number of incident tracks with a high clustering of damage around each individual track, this may obscure the potential for inter-track interactions.

This can be uncovered in mixed-field exposures, which expose cells to X-rays followed by alpha particles or vice-versa, at varying time intervals, shown in the bottom panels of Fig. 3. Both combinations show clear SLD repair, regardless of whether the X-rays or alpha particles are delivered first. This indicates that biologically relevant quantities of SLD are both induced and repaired by alpha particle exposures. In both cell lines, at late time points irradiations where X-rays were delivered second had slightly but statistically significantly lower survival than where alpha particles were delivered second (p < 0.01 for paired t-test across all points later than 2 h in both cell lines). This may indicate slightly less complete repair of SLD following alpha particle exposures, or be due to greater potentially lethal damage induced by the higher dose of X-rays compared to alpha particles, which is known to be possibly converted to lethal damage during the replating process^50^.

Notably, the rates of repair are similar in both conditions—SLD half-lives across each condition and cell line are summarised in Table 1. Although parameter uncertainties are large, repair rates for each cell line are not significantly different (and indeed, alpha particle first exposures show the lower half-lives). To confirm this, a sum-of-squares F test was used to compare fits with independent repair half-lives to one with a single repair half-life for each cell line, and showed that there was no statistically significant difference in fit quality (p > 0.25, single half-life fits illustrated in Supplementary Information). Taken together, this suggests that SLD induced by both types of radiation is of similar complexity to repair.

**Table 1:** Repair half-lives fit for each individual irradiation condition, and a shared fit across all exposures for each cell line.

Cell line repair half-life	X-ray + X-ray (min)	Alpha + Alpha (min)	X-ray + Alpha (min)	Alpha + X-Ray (min)	Shared fit (min)	p
PC-3	47 ± 6	–	44 ± 19	19 ± 32	44 ± 11	0.26
U2OS	32 ± 21	–	34 ± 15	24 ± 6	34 ± 17	0.67

Although substantial statistical uncertainty is present, repair can be seen in most cases, with similar kinetics. No repair is seen for alpha particles, so repair half-times cannot be defined. A shared fit using a single repair half-time across all irradiation conditions does not perform significantly worse than individual fits, suggesting no difference in repair for different conditions (F-test p values in final column).

Additionally, the magnitude of SLD repair seen in these combination exposures is greater than would be predicted from an exposure of 0.75 + 3 Gy of X-rays, indicating that the alpha particles have an elevated RBE for SLD. By evaluating the degree of SLD repair, it can be estimated that $RB{E}_{SLD}$ is 2.8 ± 0.9 and 3.7 ± 0.4 in PC-3 and U2OS cells, respectively. This suggests that, contrary to the apparent lack of recovery for alpha exposures alone, they generate substantially more SLD than X-rays for the same dose.

### Mechanistic modelling of mixed-field repair

**Figure 4:**
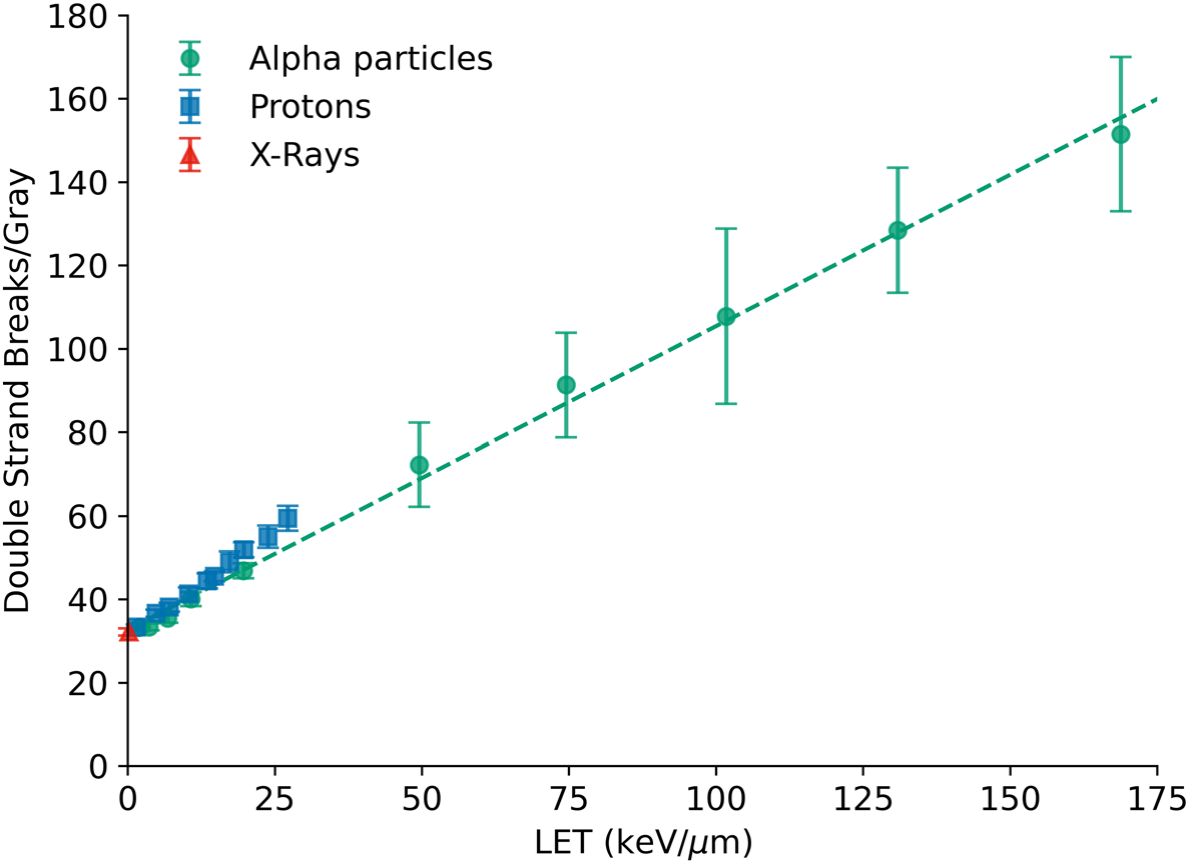
Yields of DSBs per Gray per cell predicted by a simplified nucleus model, based on clustering of SSBs deposited in proportion to delivered energy. In line with other publications, an approximately linear trend is seen between LET and DSB yield, with a degree of particle-type dependence. Points are simulation results with standard deviations, dashed line is linear fit to alpha particle DSB yield.

Assuming DSBs are the primary form of SLD induced by ionising radiation, a simple Monte Carlo model was implemented to predict differences in DSB yield as a function of LET, shown in Fig. 4. DSB yields are broadly proportional to LET, with a small particle-type dependence due to their different secondary electron spectra. These results are broadly similar to other models of high-LET $D{SB}_{RBE}$^51,52^. Based on this model, for our alpha particle exposure (with LET of 129 keV/μm), $RB{E}_{DSB}$ can be estimated as 3.67 ± 0.2, for 128.5 DSBs/Gy for alpha particles.

**Figure 5:**
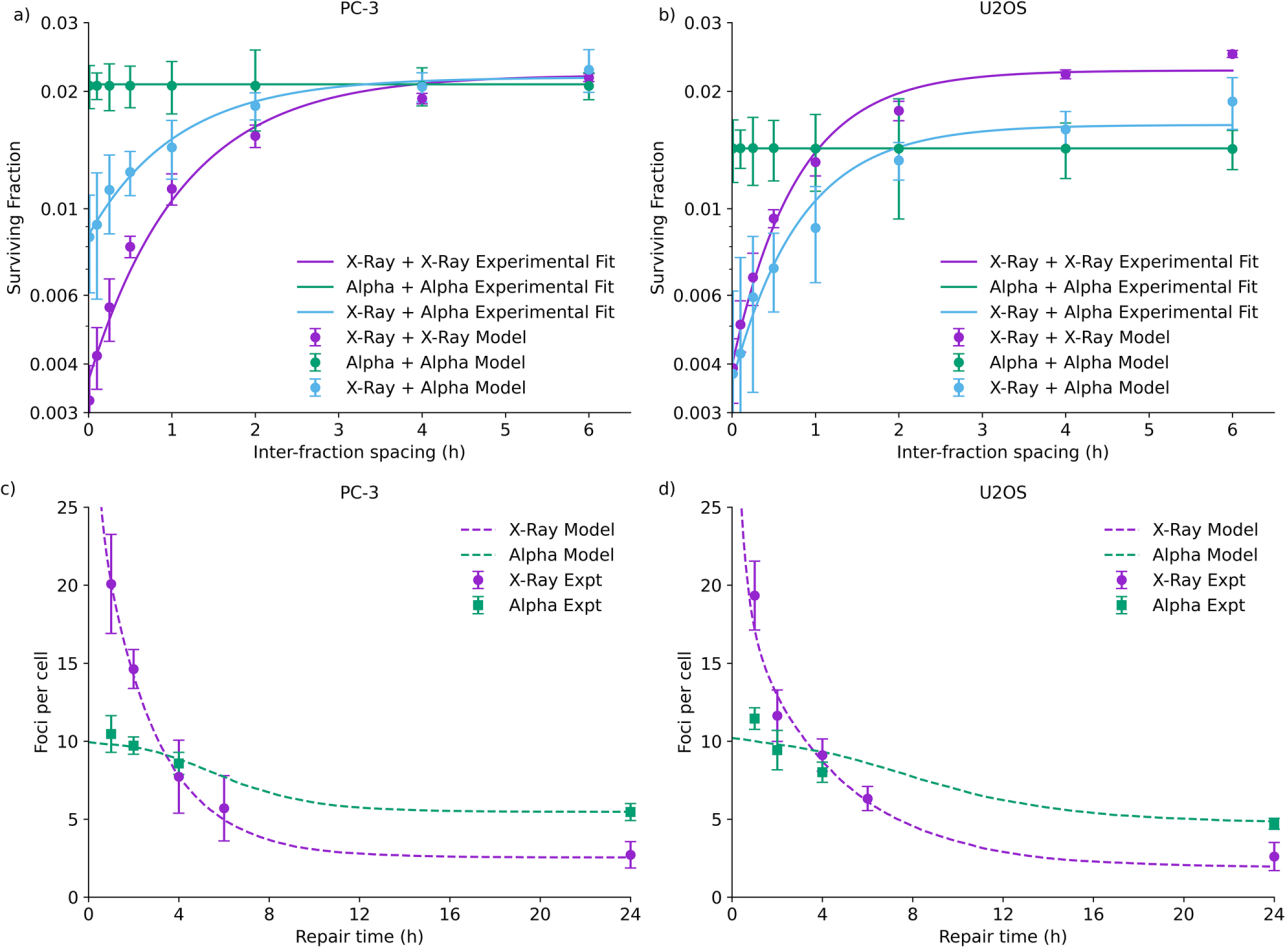
Mechanistic modelling of repair kinetics. Top: Comparison of SLDR recovery based on Medras-modelled misrepair (points) to experimental trends (lines, fits from Fig. 3). Predicted lethality is based on the number of misrepaired DSBs for each scenario as a function of fraction time. Bottom: Measured DSB damage foci (points) compared to modelled kinetics assuming that alpha particle foci represent clusters of breaks (dashed lines). This modelling approach effectively reproduces observed trends in both assays, without assuming a different in quality or repair half-life for DSBs.

By using this factor for the additional yield of DSBs generated by high-LET radiation, the Medras model can be used to predict the rate of misrepair for different exposures with different temporal separations, and thus overall trends in cell survival, illustrated in Fig. 5. These figures show that, although this model assumes no difference in the complexity and thus repair rate in DSBs induced by different types of radiation, the overall trends observed in the experimental results can be recovered. In particular, this model reproduces the observed recovery rates for all radiation combinations, as well as the lack of significant repair in the alpha + alpha irradiations, due to the highly clustered, track-based nature of alpha particle exposures. This further supports the concept that the kinetics of sublethal DSB repair are the same for both low- and high-LET exposures.

However, a constant rate of repair of DSBs seemingly conflicts with Fig. 1, which suggests that alpha-induced foci are repaired much more slowly than X-ray-induced foci. However, this may also be explained by differences in the distribution of breaks, and their impact on the ability to resolve closely overlapping foci. An analysis of a model based on this effect is shown in Fig. 5c and d, taking into account spatial clustering and its impact on resolving multiple closely-spaced foci and a DSB yield of 128.5 DSB/Gy as outlined above. This effectively reproduces the observed trend in foci repair following alpha exposures, with a greatly reduced initial rate of repair and increase in residual foci at 24 h, even though individual DSBs are repaired with the same kinetics. Best fitting parameters were 62 ± 6 and 42 ± 11 initial foci following 2 Gy X-ray exposure in PC-3 and U2OS cells respectively, 9.9 ± 0.3 and 10.2 ± 0.7 initial alpha particle damage clusters respectively, and persistent DSB rates of 0.040 ± 0.005 and 0.044 ± 0.012 respectively.

## Discussion

The impact of DSB complexity on radiosensitivity remains an outstanding question in radiobiology. A range of radiobiological models have made different predictions about the impacts of varying LET on DSB complexity, and the consequential effects of this on survival^15,16^. However, in most cases this work has focused on exposure to a single radiation quality, where it is difficult to resolve the relative contributions of DSB complexity and other factors such as DSB yield or spatial distribution. This is also the case clinically, where typically only a single type of radiation is used. As a result, a better understanding of the interactions of these radiation types would be valuable, both to improve our understanding and models of the fundamental interactions of radiation damage at higher LETs, and to support future potential research into the combination of different radiation qualities.

In this study, we investigated the interactions and kinetics of repair for different combinations of X-rays and alpha particles, representing low- and high-LET exposures. While single agent exposures aligned with expectations that SLD repair is seen for low-LET but not high-LET radiation, combining irradiation modalities revealed a substantial contribution of SLD from high-LET particles. This lack of interaction between damages is obscured in most experimental designs because at high LETs DSBs are clustered around a relatively small number of tracks. Each of these tracks individually can have a high lethality, but the probability of damages from this relatively small number of tracks interacting is much less than the probability of interactions between the more uniformly distributed damage caused by e.g. X-rays. However, by combining a small number of tracks (approximately 9 alpha particles per nucleus per Gy in this setup) with the addition of more uniformly distributed DSBs caused by low-LET radiation, the contribution of SLD can be resolved.

Two important features of SLD following alpha particle exposure can be seen in this data: firstly, $RB{E}_{SLD}$ is significantly greater than 1, comparable to that for overall cell killing; secondly, this SLD is repaired with kinetics which do not differ significantly from low-LET X-ray damage. Implementing these two observations into a mechanistic model enabled adequate reproduction of the overall trends in SLD recovery for different radiation combinations. Interestingly, the predicted SLD RBE is in line with that predicted from a mechanistic model of DSB induction, providing further support for the role of (a subset) of DSBs as the driver of SLD.

These results seem to agree with experiments in the literature which report significant interactions between X-rays and alpha particles or higher-LET ions^25–28,53,54^. Due to differences in experimental design and endpoints, it is difficult to make direct quantitative comparisons, but in all cases an overall trend of synergistic effects is observed. Furthermore, in at least some cases there is evidence for LET-dependent effects which agrees with there being an elevated RBE for DSB with increasing LET^26^.

However, some contradictory results also appear to exist, and while it is impossible to exactly compare studies, some possible explanations exist to understand these effects. Barendsen et al.^22^ reported no synergy between X-ray and alpha particles, and no recovery if those exposures were split temporally. However, in validation experiments they also reported observing no sublethal damage recovery even from fractionated X-rays, suggesting this may represent a feature of their cell lines or experimental conditions. Interestingly, both Furusawa and Durante^23^ and Phoenix et al.^24^ reported no synergistic effect from direct combinations of V79 cells to X-rays and ions or alpha particles, in different experimental setups. This may reflect a difference in responses between simultaneous and split-dose exposures, differences in the biology of V79 cells, or limitations in assay sensitivity due to the relatively high doses of alpha particles. Neither study reported split-dose or protracted dose studies to probe any temporal effects in these cellular responses, and so it is difficult to definitively say if any sublethal interactions are occurring in these systems.

A model suggesting significant SLD which is rapidly repaired also initially appears to conflict with reports in the literature of a slowing of DSB repair kinetics with increasing LET as measured by immunofluorescent and other assays^9,10,55–57^, observations which are reproduced by our own 53BP1 repair measurements in Fig. 1. Many of these apparent measurements of more persistent foci may be experimental artifacts, relating to clustering of damage and potential associated rearrangements of DNA within the nucleus. In our data a relatively simple approach which takes account of clustering of multiple DSB within observed foci can explain our observed trends in foci kinetics, suggesting that repair of individual DSBs may not be accurately reflected by foci kinetics, potentially explaining many observed differences.

The full picture of DNA repair and interactions between radiations of different qualities may be further complicated by complex evolution of foci over time. Observations made by Sollazzo et al.^29,30^, who used high-resolution and live-cell imaging of U2OS cells to map the evolution of DNA damage foci following exposure to X-rays and alpha particles, showed that foci showed a complex evolution in terms of size and distribution over time following irradiation, in some cases migrating to merge or split, changing the observed yield in a non-trivial manner. Greater understanding of the motility and distribution of such damages within the nucleus may be important to fully understand the impacts of radiations of different qualities.

This this work may be valuable in helping our understanding of the biological effects of elevated RBEs as LET increases, and their relationship with the yield and quality of DSB damage. Different DNA damage models differ significantly on both the predicted yield and complexity of DSBs for different types of radiation, as well as how these two factors combine to give rise to differences in biological response. Data like that reported in this study will provide further testing sets in less common radiation scenarios, helping to highlight the greater potential sublethal damage with increasing LET which may be obscured by other studies of fractionation which only used high-LET radiations such as alpha particles. This may also be informative for the design of future combinations of radiation qualities, as it suggests that in some cases near-simultaneous deliveries of different radiation qualities may have greater additive effects than expected. However, as the repair of this damage is rapid, protracted or well-separated exposures are unlikely to interact more than in single-quality studies.

However, there remain a number of limitations and outstanding questions which must be addressed before these interactions can be fully understood. In particular, due to the correlations between damage quantity and complexity, it is challenging to definitively rule out a contribution of complexity with indirect measurements such as these. More targeted studies, explicitly distinguishing between simple and complex damage, may help to further elucidate their dependence on LET. More generally, more detailed modelling may help to further refine model predictions and enable a fully-integrated prediction of survival, rather than the fitting-based approach used here^45,51^. Similarly, combinations of these predictions with realistic models of foci imaging techniques may help to more rigorously test the clustering-based model of DSB foci discussed in this work^58^. Finally, it is important to validate these effects in cells with differing DNA repair defects, as there is growing evidence that this affects outcomes for treatments with different qualities of radiation, such as HR defects sensitising cells to alpha particle based therapy^59^.

## Conclusions

Although single-radiation treatments suggest limited sublethal damage repair in high-LET alpha particle exposures, mixed-field exposures combining alpha particles and photons reveals a significant level of sublethal damage, which is induced with a large RBE ($RB{E}_{SLD}>2.5$ in this study), but repaired with similar kinetics to that of low-LET radiation. Taking these factors into account may be significant for the design of mixed-field treatments, and deepening our understanding of the responses to high-LET radiation.

## Supplementary Information

Supplementary Information 1.

Supplementary Information 2.

## Data Availability

All experimental data associated with this work is provided in the supplementary information.
