# Supplementary material for: High-LET radiation induces large amounts of rapidly-repaired sublethal damage
Source: Sci Rep. 2023 Jul 11;13:11198. doi: 10.1038/s41598-023-38295-3 (PMC10336062; doi:10.1038/s41598-023-38295-3)
Supplement: Supplementary file 1 — Supplementary Information 1. [file 41598_2023_38295_MOESM1_ESM.pdf]

Supplementary Data

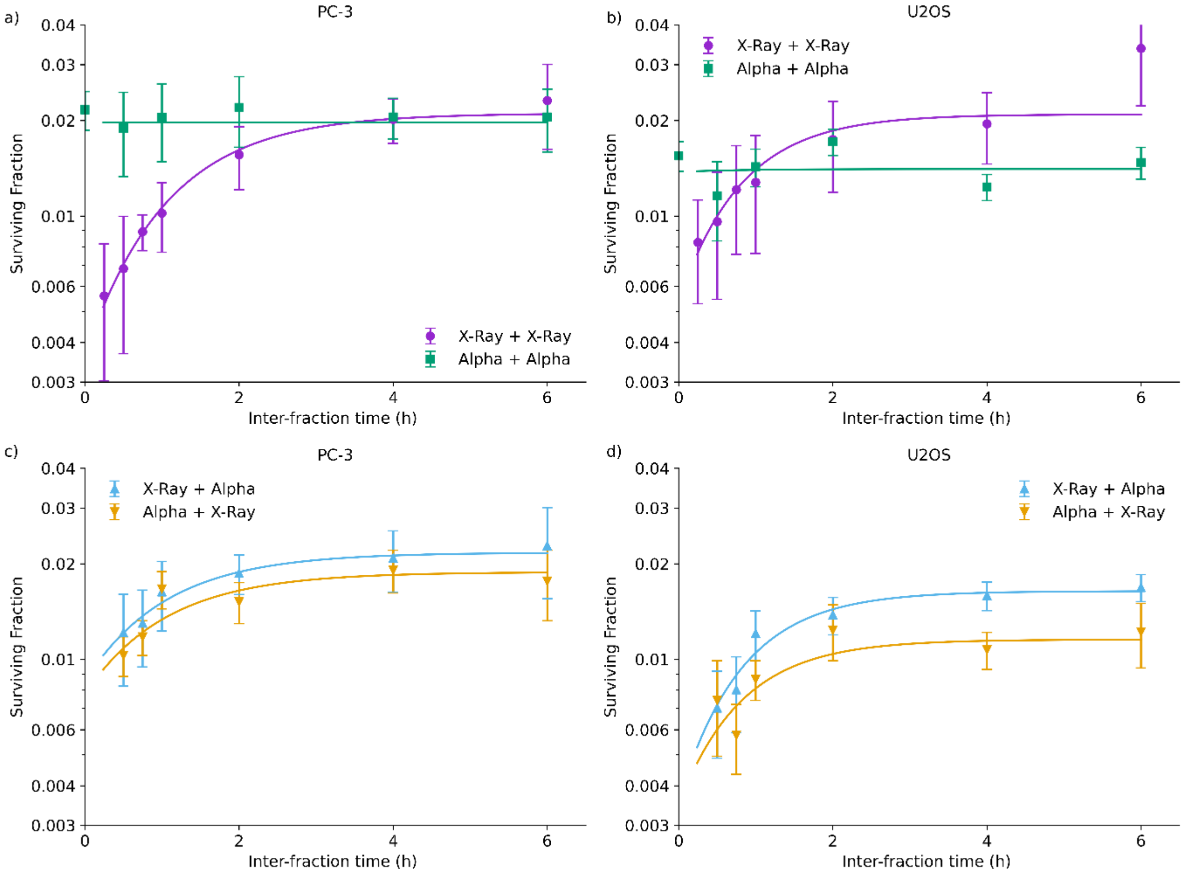

Figure S 1 Sublethal damage repair curves for PC-3 (left) and U2OS (right) cells following exposure to two identical fractions (3 Gy X-rays or 0.75 Gy alpha particles, top) or mixed exposures (bottom), as in Figure 3. Here, instead of a different repair rate for each exposure and cell line, only a single repair rate is fit for all irradiations in each cell line. This can be seen to remain in good agreement with the data, and is not statistically significantly different to the independent repair models presented in Figure 3.

		X-ray	Alpha	X-ray + Alpha	Alpha + X-ray
**PC-3**	α (Gy^{-1})	0.55 ± 0.05	2.0 ± 0.2	1.2 ± 0.2	1.4 ± 0.1
	β (Gy^{-1})	0.04 ± 0.01	0	0	0
**U2OS**	α (Gy^{-1})	0.28 ± 0.03	2.6 ± 0.2	1.7 ± 0.2	1.5 ± 0.1
	β (Gy^{-1})	0.052 ± 0.009	0	0	0

Table S 1 Linear quadratic model fit parameters for the dose response curves presented in Figure 2.
